# Label-free reflectance hyperspectral imaging for tumor margin assessment: a pilot study on surgical specimens of cancer patients

**DOI:** 10.1117/1.JBO.22.8.086009

**Published:** 2017-08-28

**Authors:** Baowei Fei, Guolan Lu, Xu Wang, Hongzheng Zhang, James V. Little, Mihir R. Patel, Christopher C. Griffith, Mark W. El-Diery, Amy Y. Chen

**Affiliations:** a Emory University School of Medicine, Department of Radiology and Imaging Sciences, Atlanta, Georgia, United States; b Georgia Institute of Technology and Emory University, Department of Biomedical Engineering, Atlanta, Georgia, United States; c Emory University, Department of Mathematics and Computer Science, Atlanta, Georgia, United States; d Winship Cancer Institute of Emory University, Atlanta, Georgia, United States; e Emory University School of Medicine, Department of Hematology and Medical Oncology, Atlanta, Georgia, United States; f Emory University School of Medicine, Department of Otolaryngology, Atlanta, Georgia, United States; g Emory University School of Medicine, Department of Pathology and Laboratory Medicine, Atlanta, Georgia, United States

**Keywords:** hyperspectral imaging, image-guided surgery, tumor margin assessment, cancer detection, image classification, image quantification, head and neck cancer, label-free, fluorescence imaging

## Abstract

A label-free, hyperspectral imaging (HSI) approach has been proposed for tumor margin assessment. HSI data, i.e., hypercube (x,y,λ), consist of a series of high-resolution images of the same field of view that are acquired at different wavelengths. Every pixel on an HSI image has an optical spectrum. In this pilot clinical study, a pipeline of a machine-learning-based quantification method for HSI data was implemented and evaluated in patient specimens. Spectral features from HSI data were used for the classification of cancer and normal tissue. Surgical tissue specimens were collected from 16 human patients who underwent head and neck (H&N) cancer surgery. HSI, autofluorescence images, and fluorescence images with 2-deoxy-2-[(7-nitro-2,1,3-benzoxadiazol-4-yl)amino]-D-glucose (2-NBDG) and proflavine were acquired from each specimen. Digitized histologic slides were examined by an H&N pathologist. The HSI and classification method were able to distinguish between cancer and normal tissue from the oral cavity with an average accuracy of 90%±8%, sensitivity of 89%±9%, and specificity of 91%±6%. For tissue specimens from the thyroid, the method achieved an average accuracy of 94%±6%, sensitivity of 94%±6%, and specificity of 95%±6%. HSI outperformed autofluorescence imaging or fluorescence imaging with vital dye (2-NBDG or proflavine). This study demonstrated the feasibility of label-free, HSI for tumor margin assessment in surgical tissue specimens of H&N cancer patients. Further development of the HSI technology is warranted for its application in image-guided surgery.

## 1 Introduction

There are ∼15.2  million new cases of cancer each year, and more than 80% of these patients will require surgery, some several times.^1^ Surgery cures ∼45% of all patients with cancer.^2^^,^^3^ To cure a cancer patient by surgery, the surgeon must remove the entire tumor at the time of the surgery. Unfortunately, up to 39% of the patients who undergo surgery leave the operating room without a complete resection due to positive or close margins.^2^^,^^4^^,^^5^ It has been reported that a complete resection is the single most important predictor of patient survival for almost all solid cancers.^6^ In breast, head and neck (H&N), lung, colon, and pancreatic cancers, complete resection is associated with a threefold to fivefold improvement in the patient survival compared to a partial or incomplete resection. ^7^^–^^10^

For cancer margin assessment, various methods have been used or are under development in order to improve the tumor resection during surgery. The visual appearance and palpation are often used by a surgeon to differentiate between malignant and normal tissue.^11^ However, this visual assessment is subjective. Intraoperative, frozen margin evaluation is commonly used to optimize surgical margin delineation at the initial surgery.^12^ Small samples from the surgical bed are selected to evaluate the presence or absence of residual cancer.^13^ However, intraoperative, frozen section diagnosis may suffer from errors that occur during sampling and histological interpretation. In addition, histological processing can take time,^14^ which is labor-intensive and prolongs the surgery time. Fluorescence-guided imaging used to navigate cancer resection has been shown to improve the number of complete resections as well as the progression-free survival.^15^^–^^21^ In most cases, fluorescence-based approaches require the injection of a fluorescence contrast agent. There are clinical needs to develop label-free imaging technology and quantification methods to aid the decision-making during image-guided surgery.

Hyperspectral imaging (HSI), originated from the remote sensing field,^22^ has emerged as a relatively new imaging modality for medical applications.^23^ This label-free imaging technology does not require a contrast agent and offers great potential for objective assessment of cancer margins. Light delivered to the biological tissue undergoes multiple scattering due to the inhomogeneity of biological structures and absorption primarily in hemoglobin, melanin, and water as it propagates through tissue.^24^^,^^25^ The absorption, fluorescence, and scattering characteristics of tissue change with the progression of diseases.^26^ Therefore, the reflected, fluorescent, and transmitted lights from tissue, where are captured by HSI, carry quantitative diagnostic information regarding tissue pathology.^26^^–^^29^ Spatially resolved spectra obtained by HSI provide diagnostic information about the tissue physiology, morphology, and composition. Recent advancements of hyperspectral cameras, image analysis methods, and computational power make it possible for many exciting applications of HSI, such as cancer detection and image-guided surgery.^23^

Previously, our group has developed a pipeline of machine-learning-based quantification methods for hyperspectral data, including image preprocessing, feature extraction and selection, and image classification, and validated these approaches in multiple preclinical animal models for both noninvasive cancer detection^30^^–^^33^ and surgical guidance.^34^ To simulate the characteristics of surgical images, our group^34^ developed a framework of hyperspectral image processing and quantification and validated the method for cancer detection during animal surgery. These preclinical studies have demonstrated that HSI has great potential to be used as a diagnostic tool for cancer detection. To translate HSI into the clinic, we designed a plot study to image surgical specimen of H&N cancer patients and implemented and validated a pipeline of quantification method to differentiate tumor from normal tissue in HSI data of human patients. The cancer detection results of HSI were compared with histopathology to determine the sensitivity and specificity.

The preliminary result of this pilot study was presented at the 2017 SPIE Photonics West Conference “Advanced Biomedical and Clinical Diagnostic and Surgical Guidance Systems XV” and was selected by the conference chairperson for submitting a full paper to the *Journal of Biomedical Optics*.^35^ In this paper, we extended the SPIE paper and added more results and discussions. This pilot study represented an important first step toward translating label-free HSI into the clinic for assessing the tumor margins of H&N cancer tissue from human patients.

## 2 Methods and Materials

### 2.1 Overview of the Study Design

**Fig. 1:**
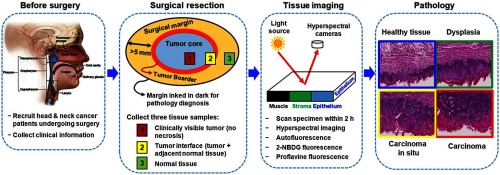
Study design for the HSI of surgical specimens of H&N cancer patients.

Figure 1 shows the study design for the HSI experiment on surgical specimens of H&N cancer patients. Before surgery, consent was obtained from the patient, and clinical information was collected. During surgery, three types of tissue, i.e., clinically visible tumor, normal tissue, and tumor with adjacent normal tissue, were collected and prepared for HSI, autofluorescence imaging, and fluorescence imaging with vital dyes, i.e., 2-deoxy-2-[(7-nitro-2,1,3-benzoxadiazol-4-yl)amino]-D-glucose (2-NBDG) and proflavine. The tissues were then processed histologically, and the pathological images were digitized and analyzed by a pathologist for validation.

### 2.2 Hyperspectral Imaging System

A Maestro (PerkinElmer Inc., Waltham, Massachusetts) imaging system was used to acquire the hyperspectral dataset. This is a wavelength-scanning system consisting of a xenon light source, a solid-state liquid crystal filter, and a 12-bit high-resolution charge-coupled device. Details regarding this system have been described in previously published papers.^36^^,^^37^ This system is capable of obtaining reflectance images over the range of 450 to 950 nm with a 2-nm increment, as well as fluorescence images under different excitation light sources.^38^

### 2.3 Surgical Specimen Collection

H&N cancer patients who underwent surgery at Emory University Hospitals Midtown were recruited into the study. All tissues were collected under the clinical protocol approved by the Institutional Review Board of Emory University. During surgery, fresh surgical specimens were collected and sent to the Pathology Department for cancer assessment. Three tissue samples, i.e., (i) clinically visible tumor, (ii) surrounding normal tissue, and (iii) tumor with adjacent normal tissue, were collected from the main specimen of each consented patient. These specimens were rinsed with cold phosphate-buffered saline (PBS) to remove excess blood on tissue.

### 2.4 Hyperspectral Image Acquisition and Quantification

Fresh surgical specimens were scanned with HSI in the following steps: (1) acquire white and dark reference hypercube before tissue imaging. White reference image cubes are acquired by placing a standard white reference board in the field of view. The dark reference cubes are acquired by keeping the camera shutter closed, (2) acquire reflectance hyperspectral images of the specimen from 450 to 900 nm with 5-nm intervals, (3) acquire autofluorescence images of the specimen with blue excitation at 455- and 490-nm long-pass emission filter. (4) Acquire fluorescence imaging with 2-NBDG (Cayman Chemical, Ann Arbor, Michigan) using blue excitation and 490 long-pass filter. For 2-NBDG imaging, the specimen was washed once after incubation of tissue in a 160  μM solution of 2-NBDG in 1× PBS for 20 min at 37°C as described in Ref. 39 and (5) similarly, acquire fluorescence imaging with proflavine (Sigma Aldrich, St. Louis, Missouri) using the same imaging setting as described in step 4). After 2-NDBG imaging, the specimen was washed once and then incubated in a 0.01% w/v solution of proflavine in 1× PBS for 2 min at the room temperature for proflavine imaging.

**Fig. 2:**
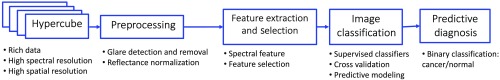
Flowchart of the machine-learning-based quantification pipeline for hyperspectral images.

Figure 2 showed the flowchart of the machine-learning-based quantification approach for hyperspectral images. In this pilot study, the steps of preprocessing, feature extraction, and image classification were implemented and validated for cancer detection in surgical specimens. To establish the histopathology gold standard, the tissue samples were histologically processed with hematoxylin and eosin (H&E) staining and pathology slides were digitally scanned, and a clinically experienced pathologist outlined the tumor border on the digitized images for the validation of the HSI classification. HSI images, autofluorescence images, and fluorescence images were manually aligned with the H&E images to map the tumor region with a software system (Analyze, AnalyzeDirect, Inc.).

### 2.5 Hyperspectral Data Normalization

The purpose of data normalization was to remove the spectral nonuniformity of the illumination device and the influence of dark current. The raw data are normalized using the following equation:

$$I_{\text{reflect}}(\lambda )=\frac{I_{\text{raw}}(\lambda )-I_{\text{dark}}(\lambda )}{I_{\text{white}}(\lambda )-I_{\text{dark}}(\lambda )},$$

where $I_{\text{reflect}}(\lambda )$ is the calculated normalized reflectance value at the wavelength λ, $I_{\text{raw}}(\lambda )$ is the intensity value of the sample pixel, and $I_{\text{white}}(\lambda )$ and $I_{\text{dark}}(\lambda )$ are the corresponding pixel intensities from the white and dark reference images at the wavelength λ, respectively.

### 2.6 Glare Detection and Removal

Glare regions are formed due to specular reflection from the moist tissue surface and do not contain useful diagnostic information regarding the tissue. Similar to the method we reported in Ref. 34, the glare detection method includes the following steps: (1) calculate the total reflectance of each pixel within a hypercube to form one reflectance image; glare pixels have higher total reflectance than normal pixels. (2) Compute the intensity histogram of this image, fit the histogram with a log logistic distribution, and then experimentally identify a threshold that separates glare and nonglare pixels.

### 2.7 Hyperspectral Image Classification

We analyze the spectral data in order to classify each pixel into normal or cancer tissue. To reduce the computational time without reducing the accuracy, spectral curves were averaged in nonoverlapping blocks of 5×5 in order to yield a spectral signature per block. All of the spectral information available in the hyperspectral data was utilized. Blocks containing glare pixels were excluded from the classification process. Each block was assigned a label as cancerous or normal.

For each patient, we used the images of the tumor and normal tissue to train the classification algorithms and then used the tumor with adjacent normal tissue to test the performance of the classification model. In other words, the classification method built the training model using the spectral features extracted from the tumor and normal tissue, and the model was then evaluated on the tumor–normal interface tissue of the same patient. We applied ensemble linear discriminant analysis as the classifier using MATLAB^®^ (MathWorks, Natick, Massachusetts).^40^

### 2.8 Pathology Validation

We used the pathology images of the same surgical specimen to validate the cancer detection using hyperspectral image classification. On the digitized, H&E-stained, pathology slides, the tumor margin was outlined by an experienced pathologist specialized in H&N cancer. To reasonably assess the performance of the classification, we chose the regions of interest where the tumor or normal tissue had been histopathologically confirmed by the pathologist.

### 2.9 Performance Metric

The sensitivity and specificity of the classifiers for each patient were calculated based on the number of correctly classified tumor and normal pixels/blocks of all the specimens belonging to this patient. We also calculated how many normal pixels/blocks were correctly classified for a normal specimen and how many tumor pixels/blocks were correctly classified for a tumor specimen as well as the sensitivity and specificity on a tumor–normal interface specimen for each patient. We evaluated the performance of the hyperspectral image classification using the areas under the curve (AUC), accuracy, sensitivity, and specificity, as defined in the following equations [true negative (TN), true positive (TP), false positive (FP), and false negative (FN)]:

$$\text{Accuracy}=\frac{\mathrm{TP}+\mathrm{TN}}{\mathrm{TP}+\mathrm{FP}+\mathrm{FN}+\mathrm{TN}},\;\text{Sensitivity}=\frac{\mathrm{TP}}{\mathrm{TP}+\mathrm{FN}};\;\text{specificity}=\frac{\mathrm{TN}}{\mathrm{TN}+\mathrm{FP}}.$$

## 3 Results

**Fig. 3:**
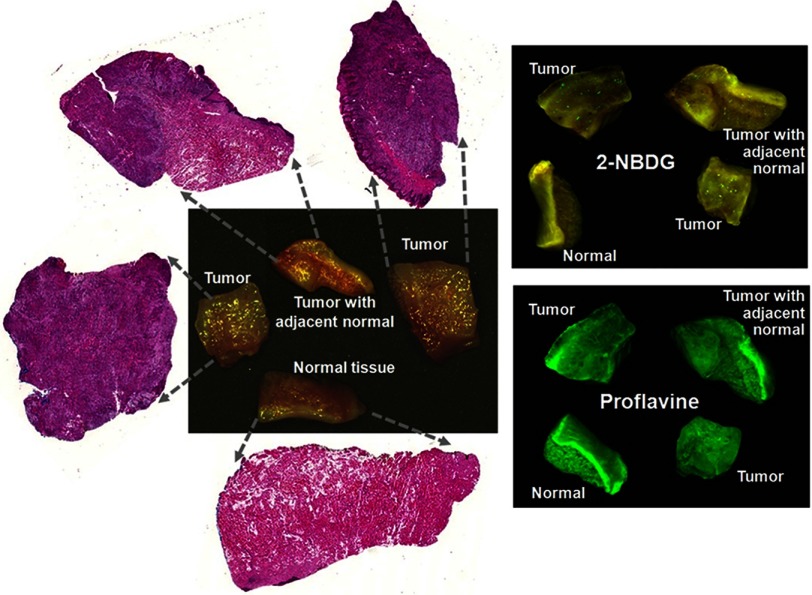
Surgical specimens of tumor, normal tissue, and tumor with adjacent normal tissue from a tongue cancer patient. Left: tissue and corresponding histological slides. Right: 2-NBDG and proflavine fluorescence images for each tissue.

Fresh surgical tissue specimens were collected from 16 H&N cancer patients. The characteristics of these patients were listed in Table 1. These patients included seven with oral cancer, one with maxillary sinus cancer, five with thyroid cancer, one with parotid cancer, and two with larynx cancer. As described above, we collected three types of tissue specimens from each human patient, which included (i) clinically visible tumor tissue without necrosis, (ii) normal tissue, and (iii) tumor with adjacent normal tissue at the tumor–normal interface. Figure 3 shows the three tissue specimens and their corresponding histological slides.

**Table 1:** Patient characteristics.

Patient	Age	Gender	Race	Tumor site	Histologic type
1	55	F	White	Tongue	Squamous cell carcinoma
2	43	M	White	Tongue	
3	67	F	White	Tongue	
4	53	M	White	Mandible	
5	76	M	Indian	Gingiva	
6	51	F	White	Floor of mouth	
7	57	M	White	Floor of mouth	
8	73	F	White	Maxillary sinus	
15	57	M	African American	Larynx	
16	69	M	African American	Larynx	
9	69	M	African American	Thyroid	Papillary thyroid carcinoma
10	59	M	Asian	Thyroid	
11	24	F	Indian	Thyroid	
12	37	M	Indian	Thyroid	
13	30	F	African American	Thyroid	
14	39	M	African American	Parotid	Pleomorphic adenoma

Note: Patients 1 to 8, 15, and 16: squamous cell carcinoma; patients 9 to 13: papillary thyroid carcinoma.

Using the reflectance spectra from HSI, the HSI method was able to distinguish between cancer and normal tissue of the oral cavity with an average accuracy of 90%±8%, sensitivity of 89%±9%, and specificity of 91%±6%. For tissue specimens from the thyroid, the method achieved an average accuracy of 94%±6%, sensitivity of 94%±6%, and specificity of 95%±6%. As shown in Table 2, HSI outperformed autofluorescence imaging, 2-NBDG, and proflavine fluorescence imaging for both cancer sites.

**Table 2:** Classification performance of HSI, autofluorescence imaging, and fluorescence imaging with 2-NBDG and proflavine.

Cancer site	Imaging method	AUC	Accuracy (%)	Sensitivity (%)	Specificity (%)
Oral cavity	HSI	0.94±0.06	90±8	89±9	91±6
	Autofluorescence	0.83±0.19	80±18	78±21	86±14
	2-NBDG	0.86±0.16	83±15	81±19	85±11
	Proflavine	0.72±0.25	70±21	71±20	70±22
Thyroid	HSI	0.98±0.03	94±6	94±6	95±6
	Autofluorescence	0.74±0.33	70±34	76±23	77±35
	2-NBDG	0.80±0.20	76±20	75±22	79±19
	Proflavine	0.86±0.16	82±16	79±18	85±13

**Fig. 4:**
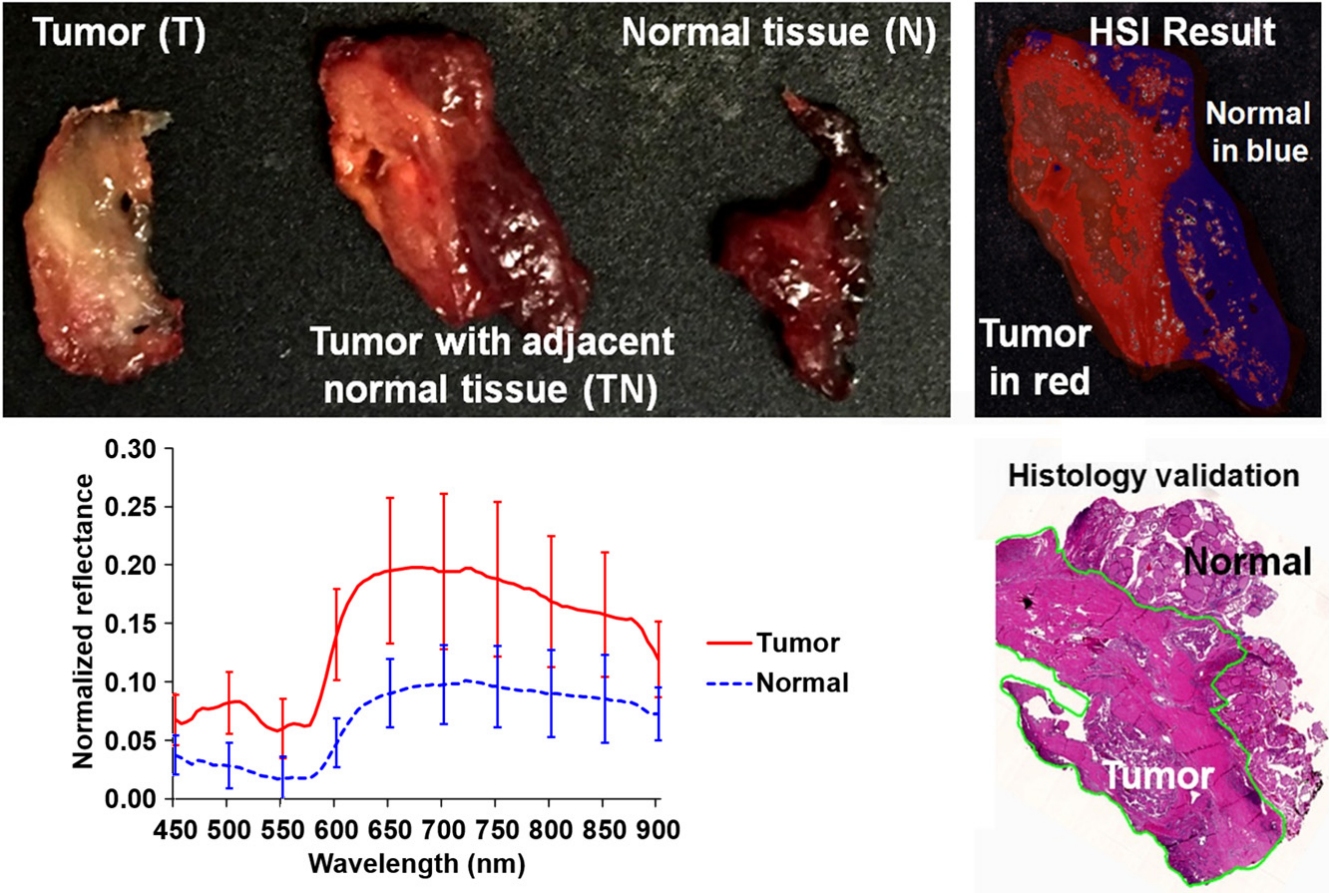
Tumor margin detection of surgical specimens from an H&N cancer patient. After hyperspectral image acquisitions, the tissue was processed histologically, and tumor margins were outlined on the pathology image (bottom right) by a pathologist (J.V.L.), which was used to validate the results of the classification (top-right). The average spectral curves are shown at the bottom left for each type of tissue, i.e., tumor, normal, and tumor with adjacent normal tissue.

Figure 4 shows the photographs of the tumor and normal tissue as well as the tumor with adjacent normal tissue for a typical case. The three types of tissue demonstrate different spectral curves. The tumor margin, as assessed by the classification method, was close to that of the histological image outlined by the pathologist.

## 4 Discussion

In this study, we reported regarding automated tissue classification methods that use the spectra from 450 to 900 nm to extract diagnostic information. Each hyperspectral image contains more than two million reflectance spectral signatures. The reflectance spectra capture the alteration of absorption and scattering properties of the tissue associated with malignant transformation. Molecular fingerprinting based on inverse modeling of reflectance spectra may shed new light on our understanding of cancer biology.

Although frozen section diagnosis is commonly used to guide surgical resection during surgery, it only samples a small portion of tissue in the resection area, which may lead to underestimation and does not guarantee margin-negative resection. In addition, this procedure is time-consuming and labor-intensive. HSI is a wide-field imaging modality that can cover a large field of view and can, therefore, provide rapid assessment of complete resection margins.

In this surgical specimen study, the label-free, HSI was superior to autofluorescence imaging or fluorescence imaging with vital dye (2-NBDG or proflavine) for the detection of H&N cancer. A recent study showed that wide-field fluorescence imaging with 2-NBDG can accurately distinguish the pathologically normal and abnormal biopsy tissue of H&N cancer patients.^41^ Proflavine has also been used for distinguishing between normal and neoplastic mucosa in the H&N.^42^ We previously demonstrated the utility of HSI for H&N cancer detection in a subcutaneous cancer animal model^31^^,^^34^ and a chemically induced oral cancer model.^43^ One important advantage of HSI is that it does not require the use of an exogenous contrast agent. Therefore, this noninvasive imaging technology can be rapidly translated from *ex vivo* tissue specimens to *in vivo* human studies, such as in clinical trials of hyperspectral, image-guided surgery.

In this study, we used the tumor and normal tissue from the same patient to train the classification and then to classify the tumor tissue with adjacent normal tissue. This approach provides reliable results and high accuracy for differentiating tumor from normal tissue. This approach is useful during surgery as this technology is helpful to the surgeon for differentiating the tumor margin while the clinician normally is aware of the tumor core but is not certain regarding the boundary of the tumor. In the future, we will test another approach that uses different patients’ data to train the classification and then test the method on a new patient. This requires a large database and we are currently collecting tissue from more patients. The combination of the two approaches may be able to provide a useful tool for the surgeon to achieve complete resection and thus improve both the patient survival and outcome.

## 5 Conclusion

HSI is an emerging imaging modality for medical applications. This label-free, HSI technology does not require a contrast agent and offers great potential for cancer detection and image-guided surgery. Hyperspectral large data contain both spatial and spectral information. Our hyperspectral image quantification tools are able to distinguish cancer from normal tissue in fresh surgical specimens of H&N cancer patients. Further development of the HSI technology is warranted for its application in image-guided surgery.
